# Assessment of Linac photon dose delivery on Cherenkov production

**DOI:** 10.1016/j.phro.2026.101083

**Published:** 2026-09-15

**Authors:** Aubrey E. Parks, Wesley S. Culberson, Brian W. Pogue

**Affiliations:** a Department of Medical Physics, University of Wisconsin-Madison, Madison, WI, USA; b Thayer School of Engineering at Dartmouth, Hanover, NH, USA

**Keywords:** Cherenkov, Cerenkov, radiation therapy, biomedical optics, IMRT

## Abstract

**Background and Purpose:**

Cherenkov imaging has emerged as a promising real-time optical dosimetry modality for treatment visualization and delivery verification. However, the dependence of Cherenkov emission on photon field size and beam-quality variations remains insufficiently characterized, particularly for highly modulated small-field delivery techniques. This work investigated whether field-size–dependent changes in dose delivery affected the production of Cherenkov photons.

**Material and Methods:**

Cherenkov emission from a tissue-equivalent optical phantom was imaged using an intensified complementary metal-oxide-semiconductor (CMOS) camera for rectangular photon fields between 0.5 × 5.0 cm^2^ and 15.0 × 5.0 cm^2^. Monte Carlo simulations using the Tool For Particle Simulation (TOPAS) framework quantified field-size–dependent changes in incident beam energy, contaminant particle spectra, secondary particle generation, and angular transport to Cherenkov production. Contributions from photons, electrons, positrons, and contaminant particles were evaluated alongside the angular distributions of Cherenkov-producing charged particles.

**Results:**

Measured Cherenkov emission per unit dose decreased nonlinearly with decreasing field size, consistent with disruption of optical equilibrium driven by tissue scattering and absorption effects. Simulations demonstrated reduced field sizes increased mean incident photon and contaminant electron energies but produced only a modest change in the energy of Cherenkov-generating particles, confirming that Cherenkov yield remains strongly dose-correlated. Secondary electrons generated within tissue dominated Cherenkov production across all field sizes, while secondary electrons originating in the accelerator head contributed measurably only in superficial regions of large fields.

**Conclusions:**

Field size dependent Cherenkov response reflects changes in particle and optical transport, highlighting considerations for Cherenkov imaging of highly modulated treatments.

## Highlights

• Cherenkov emission correlates strongly with dose across clinical field sizes.
• Small fields reduce Cherenkov nonlinearly, mainly from lost optical equilibrium.
• Beam hardening and contaminants cause minimal change in Cherenkov-particle energy.
• Secondary electrons dominate Cherenkov; contaminants matter only near the surface.
• These findings support quantitative Cherenkov dosimetry in IMRT/VMAT.

## Graphical abstract

**Figure:**
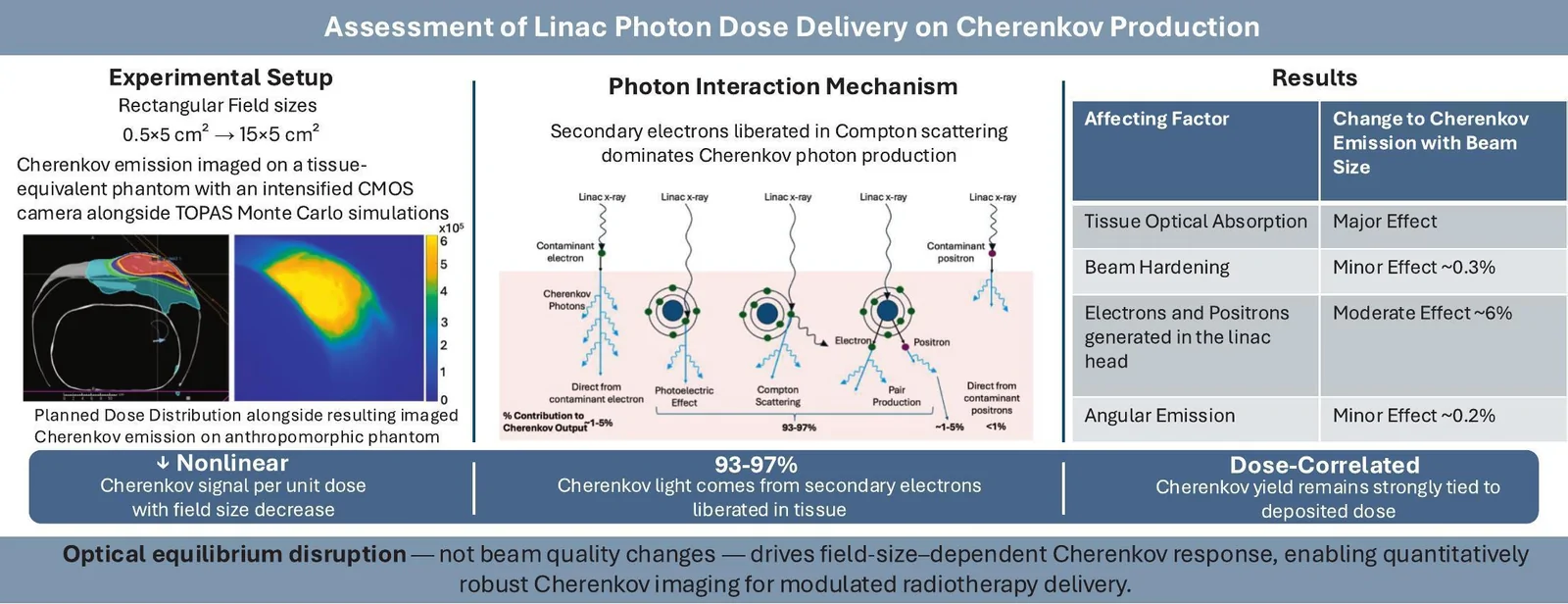

## 1 Introduction

**Fig. 1:**
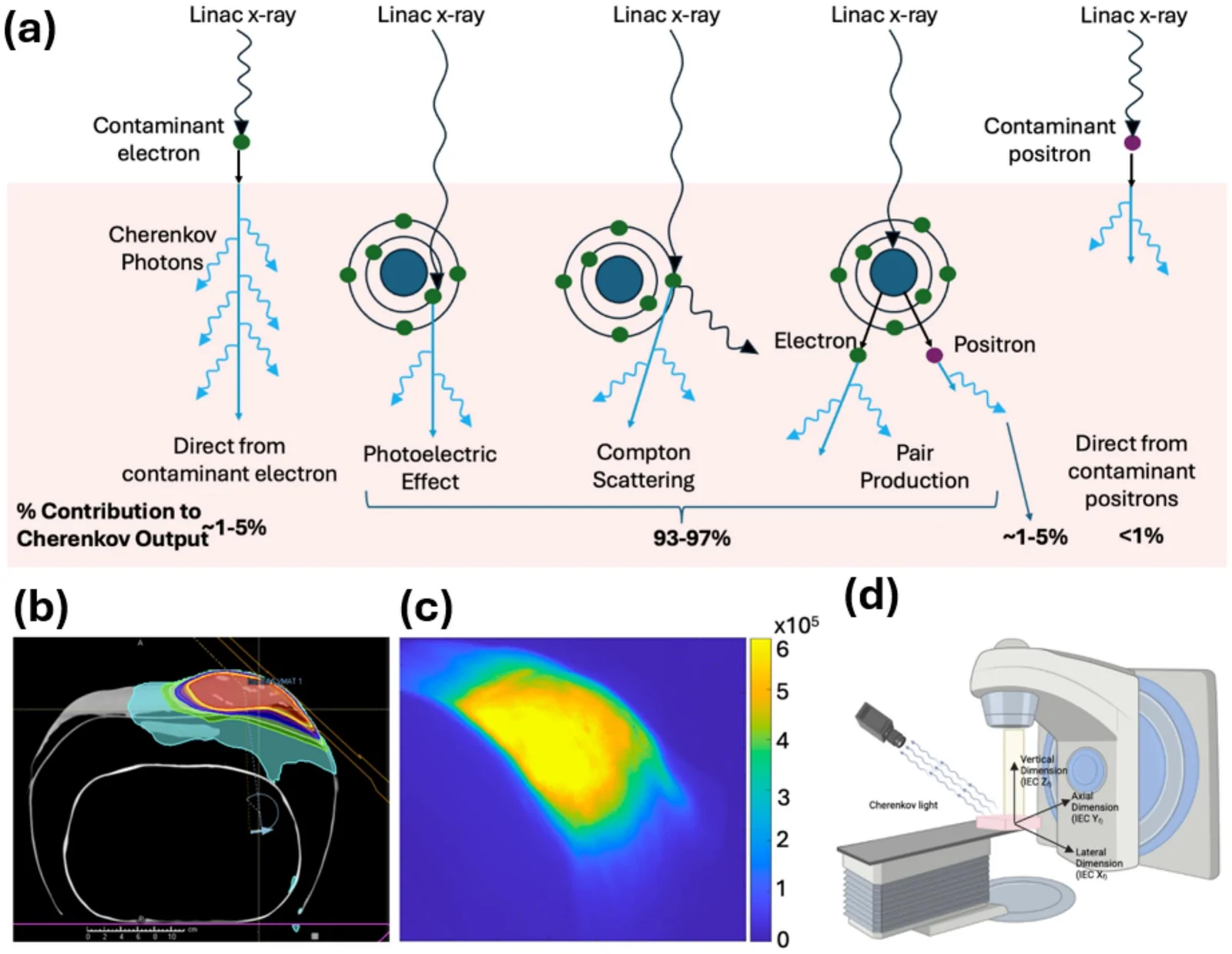
(a) Cherenkov photons are generated through the same processes of dose deposition from secondary electron soft collisions; electrons are primarily created from the photoelectric effect, Compton scattering, and pair production, with additional contributions from contaminant electrons and positrons. (b) Example planned IMRT dose distribution from RayStation TPS on an anthropomorphic body phantom in the transverse direction. (c) Resulting cumulative image of detected Cherenkov image from dose planned in (b) using the imaging setup in (d). (d) Setup of projection view imaging. The camera was mounted to the ceiling and imaged downward at the surface of an optical tissue phantom with scattering and absorption similar to soft human tissue.

In megavoltage (MV) radiation therapy, Cherenkov photons are generated as a byproduct of dose deposition, emitted from patient tissue, and detected using specialized imaging systems [1], [2], [3], enabling real-time visualization of the beam's entrance and exit on the patient. As high-energy photons propagate through tissue, interactions via the photoelectric effect, Compton scattering, and pair production deposit energy and liberate secondary electrons and positrons [4], [5], [6], [7]. When they exceed the Cherenkov threshold energy, they emit Cherenkov radiation as part of their energy transfer to the tissue, illustrated in Fig. 1a.

Because Cherenkov emission arises from the same secondary charged particles that deposit dose, its intensity is often assumed to be proportional to dose. In general, increased dose deposition leads to increased Cherenkov photon production [8], [9]. However, Cherenkov emission is not strictly proportional to dose and depends on external-beam parameters. The physical processes governing dose delivery from a linear accelerator (linac) are complex and may independently influence Cherenkov production and emission. Variations in beam characteristics, including incident average beam energy, contaminant particles, secondary electrons generated in the linac head, and the angular distribution of incident particles, can differ substantially between large and small fields [10], [11]. Additionally, tissue optical properties distort the Cherenkov signal through scattering and absorption [5], [12], [13], [14], [15]. Because Cherenkov emission is sensitive to both energy and angular characteristics of charged particles, as well as tissue optical absorption and scattering, variations in beam delivery may produce measurable differences in Cherenkov output that are not predicted by dose alone.

Therefore, a systematic investigation of how linac delivery parameters influence Cherenkov production in biological tissue is needed. Examining differences between large and small field irradiation provides further insight into the relationship between beam delivery and Cherenkov emission. This is particularly important in the context of intensity-modulated radiation therapy (IMRT), where dynamic and very-small field sizes are routinely employed. A comprehensive understanding of these effects is essential for the accurate interpretation and clinical application of Cherenkov imaging.

## 2 Materials and methods

### 2.1 Tissue-like experimental data

To investigate changes in Cherenkov emission intensity from small fields with tissue optics, RayStation Treatment Planning System (TPS) generated 20 treatment plans with field sizes ranging from 0.5 to 15 cm in the lateral direction, defined as the IEC 61217 X_f_ dimension, with a constant 5.0 cm in the axial direction, defined as the IEC 61217 Y_f_ dimension. Field sizes were collimated using both the jaws and MultiLeaf Collimators (MLCs). Plans were prescribed a uniform delivery of 1000 monitor units (MU) from a 6MV beam and delivered to a 22 × 22 × 3 cm^3^ phantom composed of 1% blood (Lamphire Biologicals, Pipersville PA), and 1% intralipid (Sigma-Aldrich, Inc., St Louis MO), which mimics tissue optical absorption and scattering [16]. The phantom dose grid had a 2 × 2 × 2 mm^3^ voxel resolution to ensure accurate dose calculations for the smallest fields. Irradiations were delivered using a Varian TrueBeam™ linac (Varian Medical Systems, Palo Alto, CA), and Cherenkov emission images were acquired for each field using a projection-view imaging system equipped with an intensified CMOS camera (C-Dose Research, DoseOptics LLC, Lebanon, NH). The experimental setup is shown in Fig. 1d. Emission intensity was quantified within a 0.3 × 0.3 cm^2^ region of interest (ROI) centered in each field, constrained by the smallest field dimension and selected to be larger than the dose grid to improve the signal-to-noise ratio (SNR) of the data. This measured intensity was compared to the total dose deposited within the superficial 5 mm of the same region, calculated through summation of point doses in RayStation TPS. This methodology enabled evaluation of the reduction in detected Cherenkov emission per unit dose as field size decreased, while accounting for the influence of tissue-mimicking optical properties.

### 2.2 Monte Carlo simulations

Monte Carlo (MC) simulations provide the most robust framework for isolating contributions from distinct particle sources, a task not feasible with experimental measurements alone. Due to the need for accurate modeling of both particle transport and optical photon generation, the Tool for Particle Simulation (TOPAS) was selected for this study [17]. The simulation geometry and beam model were largely based on a previously reported configuration [18], with additional custom scoring implemented to extract the data required for this analysis. An International Atomic Energy Agency (IAEA) phase space file for a Varian TrueBeam was placed above the moveable components of the linac, followed by the X-Jaws, Y-Jaws, base plate, MLCs, and the exit window. This allows the collimation for each of the field sizes with both the jaws and the MLCs, matching the experimental acquisition. The simulated water tank was placed at 100 cm SSD. The simulation was validated against the PDD of Varian Golden Beam data for the 10 × 10 cm^2^ field size.

Changes in average beam energy were evaluated by employing the built-in phase-space scorer positioned at the surface of a water-tank. Photons and electrons generated in the linac head were separated and analyzed independently to provide information on the energy distribution of the emitted radiation from the linac. To characterize the energy of Cherenkov-producing particles, a custom scorer was extended to record the energy of all particles generating Cherenkov photons within a 30 × 30 × 0.3 cm^3^ scoring volume centered on the beam axis, enabling analysis across the full lateral beam profile. The angular distributions of both charged particles and emitted Cherenkov photons were quantified within this volume by further customizing the scorer. The scoring volume encompassed the full 30 cm depth of the water tank and the entire lateral beam profile. The 0.3 cm thickness was selected to fit within the smallest field investigated while remaining comparable to the experimental scoring volume. This geometry is well suited for evaluating the angular and energy characteristics of Cherenkov photon production across the entire beam profile. These simulations were performed using 8 × 10^8^ particle histories, resulting in a 0.091% uncertainty.

The spatial origin of Cherenkov-producing particles was evaluated by scoring Cherenkov photons in voxels along the central axis. Dose deposition attributable exclusively to contaminant electrons was determined using the built-in dose-to-water scorer, combined with a custom filter to exclude electrons generated within the water phantom. The relative contributions of Cherenkov emission from secondary electrons and positrons generated in the linac head, along with the corresponding dose, were evaluated within 2 × 2 × 2 mm^3^ voxels located at depths of 1, 3, 5, 7, and 9 mm along the central axis. The small cubic scoring voxel was translated along the central axis to provide depth-resolved measurements without overlap between adjacent voxels. For example, the voxel centered at 1 mm spans 0–2 mm, while the voxel centered at 3 mm spans 2–4 mm. Within each voxel, a phase space-scorer was used to capture every Cherenkov photon that was locally created, while a dose-to-water scorer simultaneously calculated the corresponding delivered dose. The Cherenkov signal per unit dose was calculated through the ratio of the two values. This depth range is relevant for detecting Cherenkov emission exiting tissue, which is predominantly generated within the superficial ∼8 mm of tissue [4]. These simulations were executed with 1 × 10^9^ particle histories.

## 3 Results

**Fig. 2:**
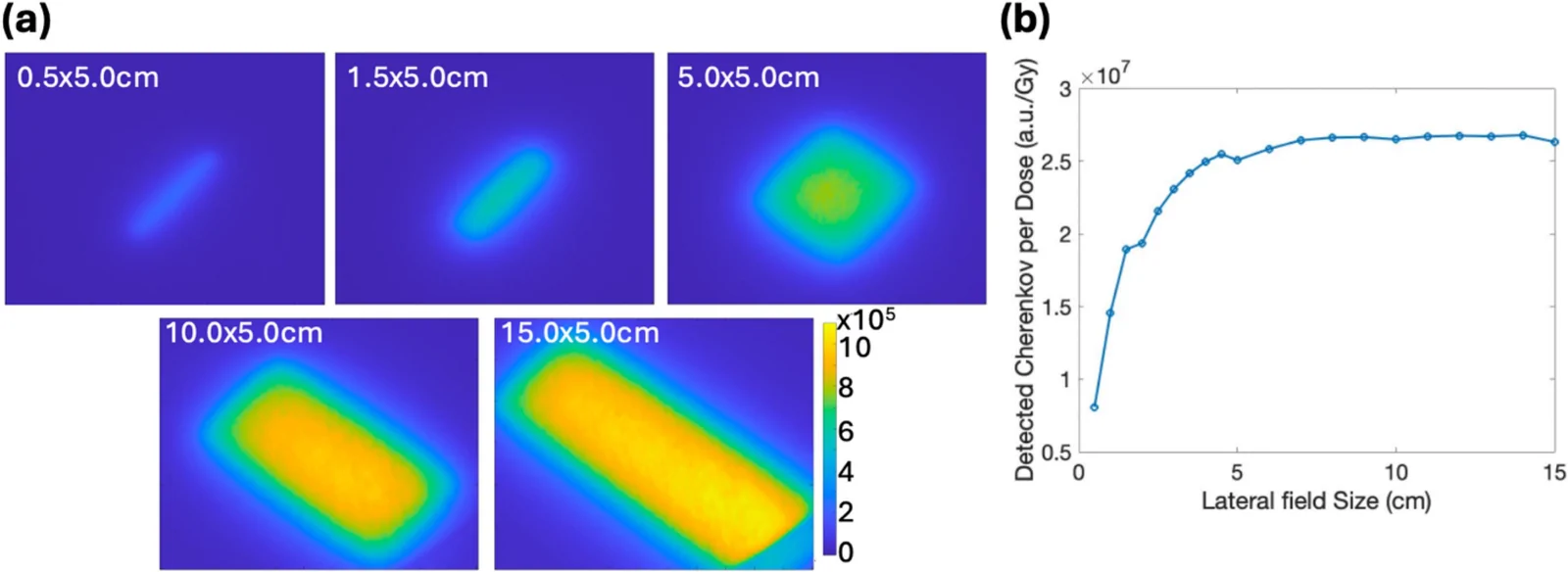
(a) Cumulative images of the detected Cherenkov signal for a sample of field sizes from experimentally acquired data. (b) The quantified detected average Cherenkov signal per unit dose is plotted for the field sizes in a small ROI at the center of each field.

Fig. 2a displays the detected Cherenkov images and detected Cherenkov signal per unit dose, calculated by RayStation TPS, which is then evaluated in Fig. 2b for experimentally acquired data. The Cherenkov signal per unit dose remains relatively constant for the largest fields but slowly declines as the field size decreases. The detected intensity is within 95% of the 10 × 5 cm^2^ field until the 4 × 5 cm^2^ field, and within 90% of the 10 × 5 cm^2^ field until the 3 × 5 cm^2^ field. The smallest field can only detect 30.5% of the 10 × 5 cm^2^ field intensity.

**Fig. 3:**
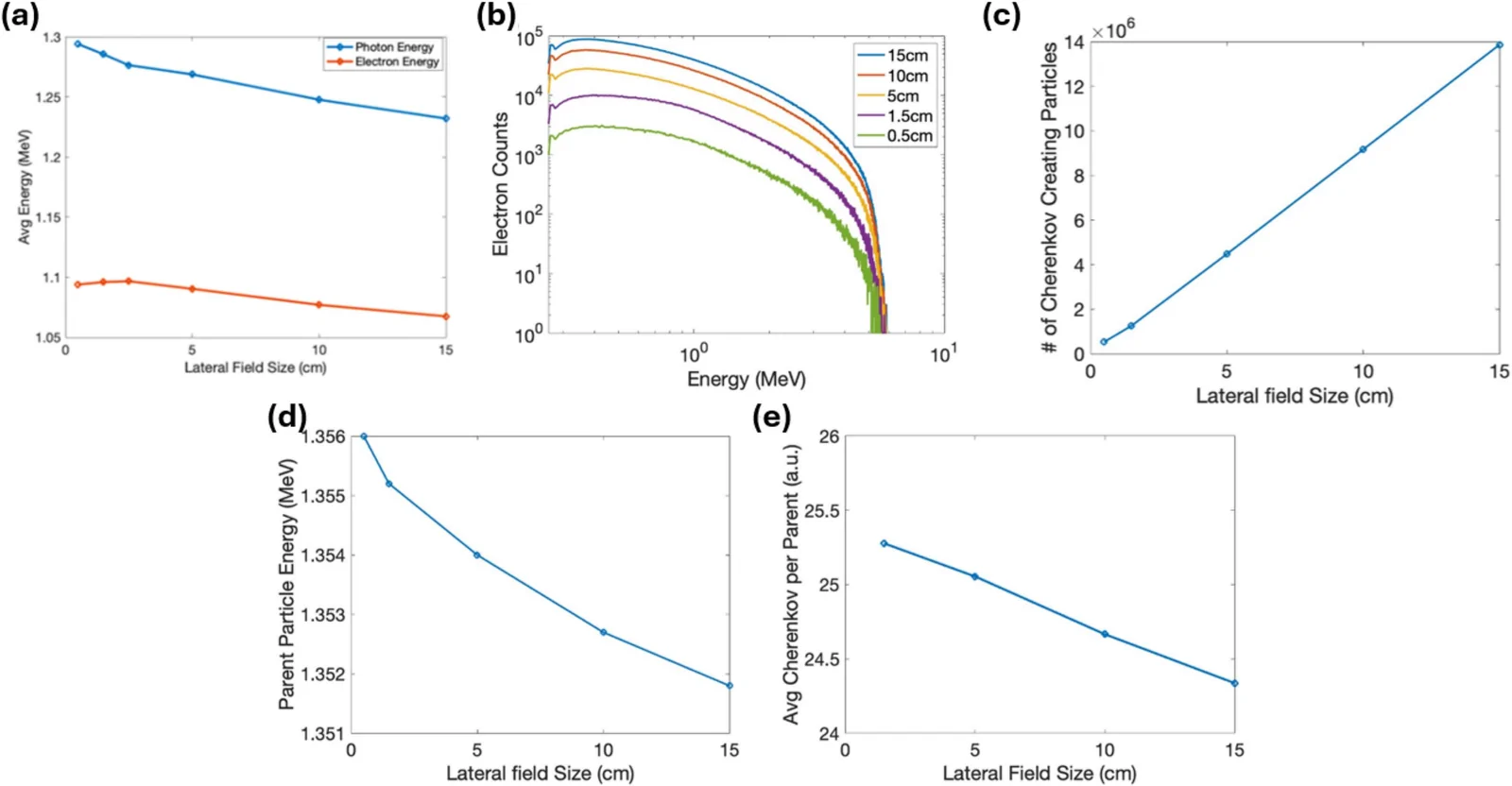
MC simulation results showing (a) The average energy of a photon beam increases 5.0% between the largest and smallest fields, and the average contaminant electron energy increases by 2.5%. (b) Energy spectra of the Cherenkov-producing particles for each of the varying field sizes. (c) The number of Cherenkov-producing particles compared to field size. (d) The average energy of the Cherenkov-producing particles with an average uncertainty on the mean of 0.00066 MeV. (e) The Average Cherenkov photons created per number of Cherenkov-producing particles.

As field size decreases, increased collimation leads to increases in the average incident beam energy, primarily due to changes in scatter within the linac head [11]. Smaller fields irradiate a reduced volume of the treatment head and phantom, resulting in fewer scattered, lower-energy photons contributing to the incident particle spectrum. In addition, reduced phantom scatter and loss of lateral charged particle equilibrium further modify the particle spectrum. Together, these effects produce a modest increase in the average energy of the incident particle spectrum as field size decreases. The average energies for the field sizes are summarized in Fig. 3a, revealing a 5.0% increase in incident photon energy and a 2.5% increase in incident electron energy between the smallest and largest field sizes, determined through MC simulations.

Fig. 3b shows the energy distribution of the Cherenkov-producing particles across the beam profiles at the beam center in a 3 mm width volume from MC simulations. The spectrum of Cherenkov-producing particles spans the Cherenkov threshold energy of 263 keV to just under 6 MeV, consistent with the incident beam energy. The number of Cherenkov-producing particles increases linearly with field size (Fig. 3c). The average parent energy increases moderately with decreasing field size, corresponding to a 0.3% change (Fig. 3d). The average number of Cherenkov photons produced per parent particle is roughly constant at 25, increasing moderately with a decrease in field size due to the increase in average parent particle energy with smaller fields (Fig. 3e). The smallest field size data point was excluded due to decreased statistics. Because the average distance to Cherenkov creation is larger than the width of the smallest field [18], many of the Cherenkov photons that are created from the parent particles are created outside of the scoring volume tested, reducing the statistics.

**Fig. 4:**
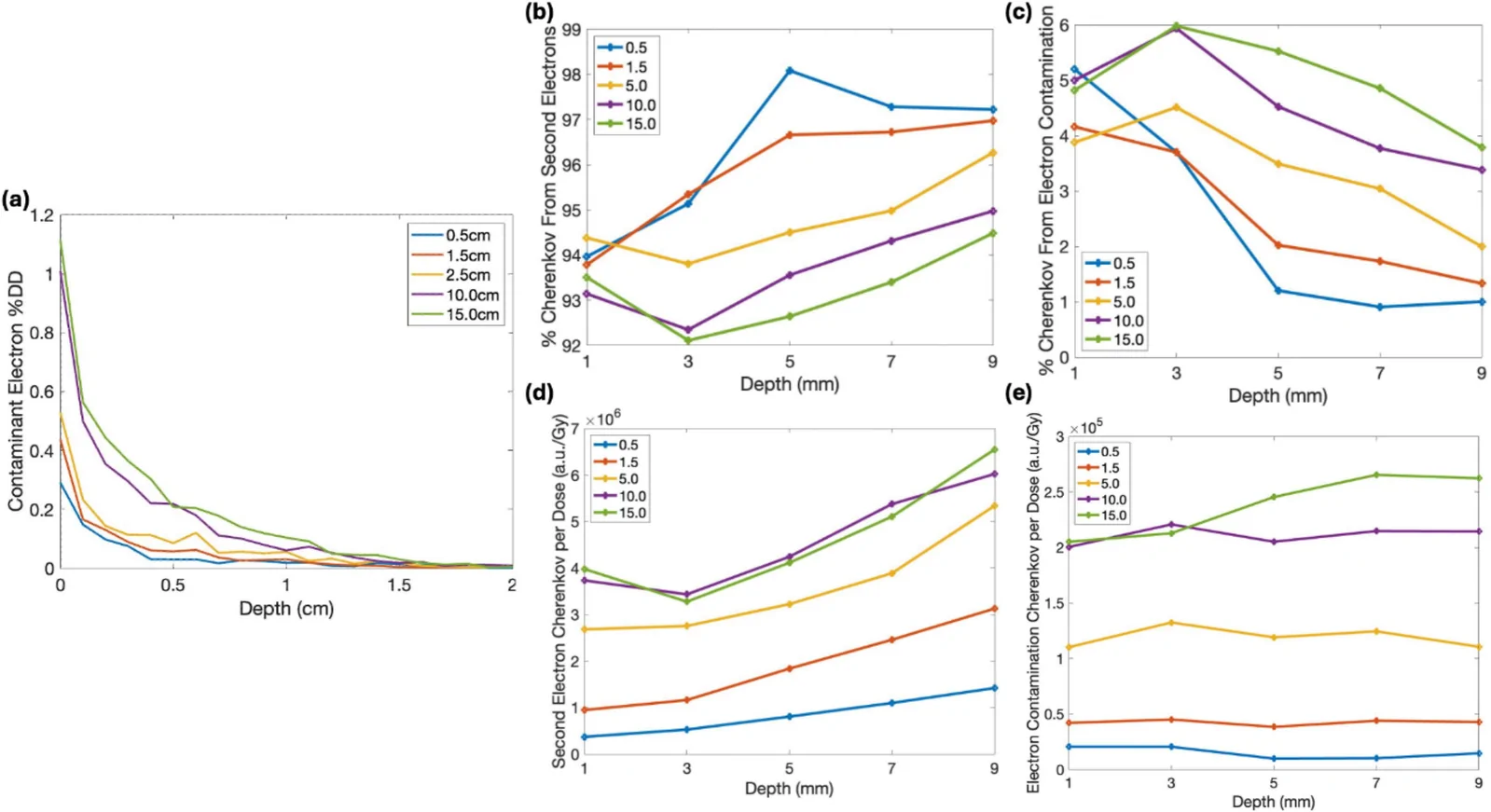
MC simulation results showing (a) Percent dose due exclusively to electron contamination. (b) Percentage of Cherenkov photons created due to secondary electrons. (c) Percentage of Cherenkov photons created in volume due to electron contamination. (d) Cherenkov photons created due to secondary electrons per unit dose in a voxel. (e) Cherenkov photons created due to contaminant electrons per unit dose in a voxel.

The composition of incident particles to the phantom surface varies with field size; larger fields exhibit increased electrons and positrons generated in the linac head compared to smaller fields. This is primarily due to enhanced interactions within the linac head, specifically within the MLCs, and the air column between the linac and the patient. These electrons play a significant role in surface dose deposition, illustrated in Fig. 4a, with elevated surface dose within the top 2 cm of tissue. MC simulations show the largest field has 1.1% of the surface dose from contaminant electrons, whereas the smallest field has only 0.3%. The larger field's contaminant electrons contribute to a further depth in the tissue than the smaller field, with the largest field contributing more than 0.1% until ∼1 cm depth, whereas the smallest field contributes more than 0.1% of the dose until 0.25 cm depth.

Because Cherenkov emission is intrinsically linked to dose deposition, variations in contaminant electron fluence influence the number of superficially generated Cherenkov photons. Fig. 4b presents the MC derived percentage of Cherenkov photons originating from secondary electrons, while Fig. 4c shows the number of Cherenkov photons produced from contaminant electrons. With reduced field size, more Cherenkov photons are produced at every depth from secondary electrons, with ∼94 to ∼98% of the Cherenkov produced from secondary electrons for the smallest field size, compared to ∼92 to ∼94% for the largest field size. The Cherenkov produced due to contaminant electrons decreases with depth as the energy from the contaminant electrons is deposited, whereas the Cherenkov produced from secondary electrons increases with depth as the cascade of secondary electrons increases. Fig. 4d and e display the number of Cherenkov photons produced per unit total dose from the secondary electrons and the contaminant electrons, respectively. When normalized to dose, there are ∼11 to ∼4 times more Cherenkov photons produced per unit dose between the smallest and largest fields from secondary electrons and ∼ 25 to ∼10 times more Cherenkov photons produced per unit dose between the smallest and largest fields due to contaminant electrons. Up to 0.25% of the Cherenkov photons are produced from contaminant positrons and up to 2% are produced from positrons created in pair production, but with no trend with changing field sizes.

**Fig. 5:**
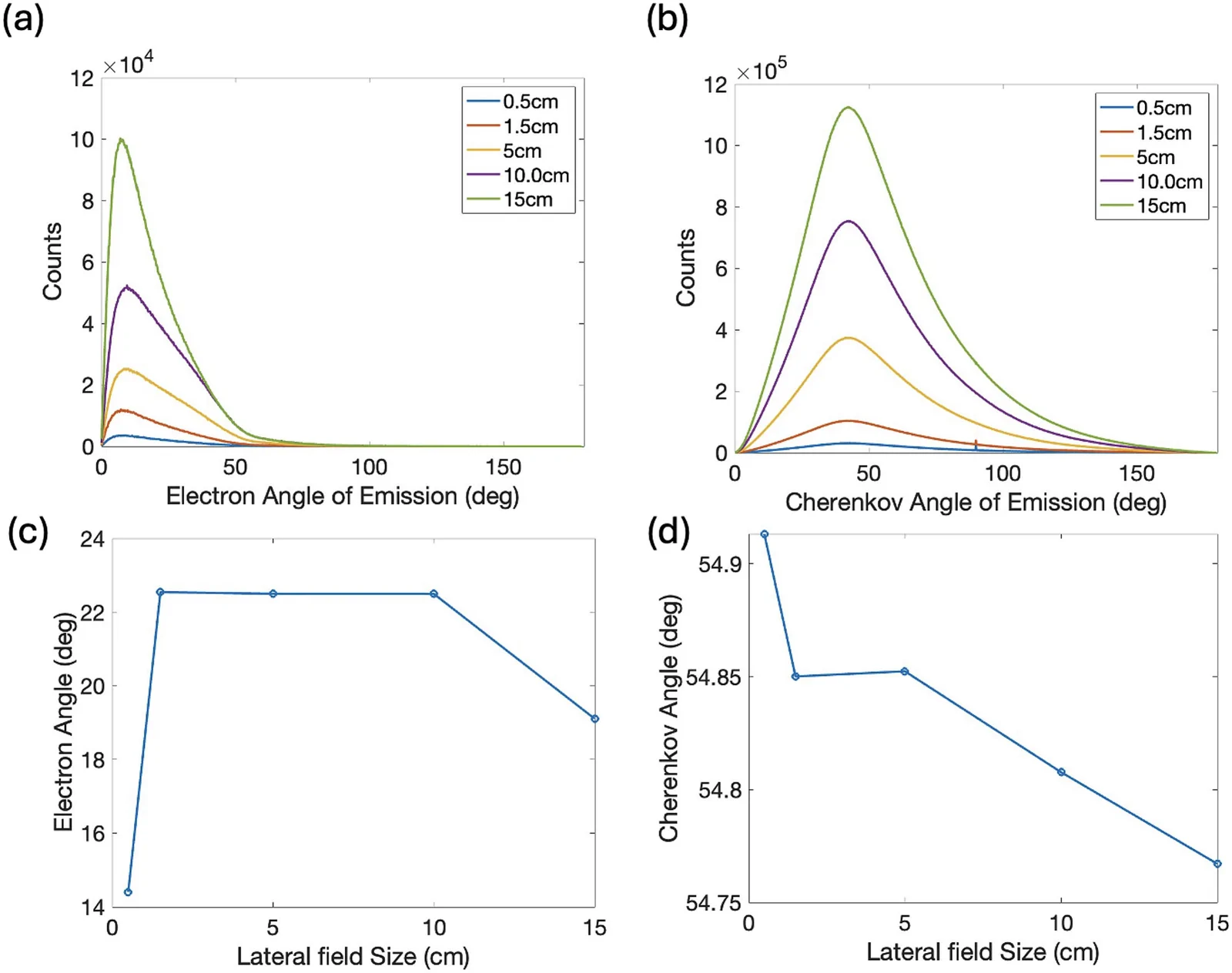
MC results showing (a) Spectrum of angular emission of the Cherenkov producing particles with respect to the negative z axis. (b) Spectrum of Cherenkov photons angular emission with respect to the negative z axis. (c) Average angle of emission of Cherenkov producing particles with an average uncertainty on the mean of 0.0020°. (d) Average angle of emission of Cherenkov photons with an average uncertainty on the mean of 0.0076°.

Fig. 5 displays the spectrum of angular creation for the incident electrons and Cherenkov photons, alongside average values, with respect to the negative z-axis, found through MC simulations. The average angle of electron creation remains relatively constant between most of the field sizes at ∼23°, but decreases to ∼14° for the smallest field size and ∼ 19° for the largest field size, indicating that there is no trend with changing field sizes. The average Cherenkov emission angle shows a general, but likely insignificant, increase from 54.7° for the largest field size to 54.9° for the smallest field size.

Major effects impacting Cherenkov production with respect to field size are summarized in Table 1.

**Table 1:** Summary of linac beam quality effects on Cherenkov production output with respect to changes in lateral beam diameter.

**Affecting Factor**	**Effect on Cherenkov Production**	**Magnitude with Beam Size**
**Tissue optical absorption**	Tissue optical absorption reduces the total signal reaching the tissue surface. Scattering increases the contribution to optical photon redistribution in tissue, increasing blur and decreasing detected signal.	**Major effect** Photon redistribution becomes relevant for the 4.0 × 5.0 cm^2^ field, compared to the 2.0 × 5.0 cm^2^ field in the absence of tissue optics [18].
**Average Beam Energy Change**	Average energy of photon & electron spectra increases due to absorption of low-energy particles in linac head. Most absorbed particles are below Cherenkov threshold, so this has a low Cherenkov production effect.	**Minor effect** ∼0.3% change in average energy of Cherenkov producing particles
**Contaminant Electrons and Positrons**	Smaller field sizes have less dose contribution from electron contamination at the surface, and they have less Cherenkov photons produced from contaminant electrons than larger beams.	**Moderate effect** ∼6% reduction in the most superficial 1 cm of tissue
**Angular Emission**	Cherenkov producing particles have a largely downward distribution, with a large angular spread avg. ∼22 degrees. This results in wide spread of Cherenkov emission angles centralized around the characteristic Cherenkov emission angle of 42 degrees.	**Minor effect** ∼0.2% change in avg. Cherenkov angle of emission with field size. No change in magnitude of Cherenkov emissions

## 4 Discussion

Interpreting Cherenkov emission as a function of beam quality parameters is complex because radiation transport occurs at both MV photon energies and optical Cherenkov energies. In tissue, optical photon transport is governed primarily by absorption and elastic scattering. Absorption attenuates the detected signal, while scattering redistributes photons, increasing the spatial spread of the light [13]. Field size further influences these optical effects, resulting in a nonlinear reduction in Cherenkov signal per unit dose. In contrast, when tissue optical properties are neglected, the reduction in Cherenkov signal per dose with is more abrupt with decreasing field size [18]. With tissue optical properties accounted for, absorption attenuates the overall signal, while scattering enhances photon redistribution and contributes to a loss of optical equilibrium at larger field sizes. As a result, in the absence of tissue optics, optical spreading significantly affects only the smallest field sizes. When tissue optical properties are included, the reduction in Cherenkov signal larger field sizes are effected. This distinction is particularly important for Cherenkov imaging during IMRT, where small treatment fields are common. In the tissue phantom, the detected Cherenkov signal per unit dose for the smallest field was only 30.5% of that measured for the largest field, demonstrating the substantial impact of tissue optics on quantitative Cherenkov imaging. Previous studies have similarly shown that tissue constituents, including oxygenated hemoglobin, deoxygenated hemoglobin, and melanin, strongly influence the detected Cherenkov signal [8], [12], [16], [20], [21]. The experimental measurements quantify the combined effects of field size and tissue optical properties on the detected Cherenkov signal. To isolate the mechanisms governing Cherenkov production, Monte Carlo simulations were performed without tissue optical properties, allowing the effects of optical transport to be separated from intrinsic changes in Cherenkov generation.

The Frank–Tamm formula (Eq. 1) describes the amount of Cherenkov radiation produced as a function of the charged particle and medium properties [19]. The variable parameters include the medium's index of refraction and the particle's velocity, and therefore its energy. In biological tissue, the refractive index is relatively constant, similar to that of water. However, higher-energy particles generate more photons than lower-energy ones. Consequently, an increase in the average particle energy could lead to a corresponding increase in Cherenkov yield.

(1) $$\frac{dN^{2}}{\mathrm{d\lambda dx}}=\frac{2\pi \alpha x^{2}}{\lambda^{2}}\left(1-\frac{1}{\beta^{2}n^{2}}\right)$$

As field size decreases, increased collimation reduces low energy scattered photons reaching the patient, increasing the average energy of the incident particle spectrum. Measurements of the particle spectra incident on the water phantom showed average energy increases of 5.0% for photons and 2.5% for electrons.

The energy spectrum of particles producing Cherenkov light changes less with field size, from ∼1.3518 MeV to ∼1.356 MeV (∼0.3% increase) in average incident particle energy, which is consistent with the results in Fig. 3, as most changes in incident particle energy occur below the Cherenkov threshold of 263 keV. Because this shift in average energy is primarily due to the loss of low-energy, sub-threshold particles, it does not produce a major change to the Cherenkov produced with changing field sizes.

As the field size increases, the Cherenkov-producing particles increase linearly, but the average Cherenkov per parent remains constant at ∼25 photons. This is consistent with previous work demonstrating that Cherenkov production per unit dose remains constant along the central axis across all field sizes [18].

A more significant contributor to field-size-dependent Cherenkov emission is the change in incident particle composition. As the field size increases, the number of contaminant particles incident on the patient also increases [20], [21], [22]. These contaminant electrons contribute substantially to surface dose. Because Cherenkov emission is superficial, the rise in contaminant electrons may lead to enhanced Cherenkov photon production because they do not rely on secondary electron interactions.

Fig. 4b and c show increasing field size yielding an ∼6% increase in the produced directly by contaminant electrons, relative to secondary electrons. When normalized to dose (Fig. 4d and e), Cherenkov photons originating from contaminant electrons remain constant across the most superficial centimeter. This is because, while the Cherenkov photon yield from contaminant electrons diminishes with depth, the dose increases within the buildup region, resulting in a balanced depth-dependent contribution. This effect is responsible for enhanced Cherenkov emission observed from electron beams, such as in Total Skin Electron Therapy (TSET) applications [23], [24], [25].

When looking at the angle of emission of Cherenkov-producing particles with respect to the negative z-axis (Fig. 5a), there is a general downward trend, with an angular spread to account for particle scatter. The average angle for the Cherenkov-producing particles is ∼23° across most fields, with reductions in the smallest and largest fields, indicating no trend with field size. Cherenkov photons have a characteristic emission angle of 42° in water [19], [26], [27]. In Fig. 5b, Cherenkov emission peaks just above 42°, with a broader angular spectrum than a single peak. The reason for this is the Cherenkov-producing particle's angular distribution because particle trajectories are not ideally aligned with the beam axis. There is a modest increase in the average angular distribution of Cherenkov photons of ∼0.2%.

Accurate patient alignment is critical and Cherenkov imaging offers a method for real-time visualization. However, without clearly understanding Cherenkov emission variations with physical dose delivery, complete signal interpretation is uncertain. The present results demonstrate that field size can influence Cherenkov emission through changes in tissue optical transport and particle characteristics, which should be considered when interpreting Cherenkov images of modulated treatments.

Tissue optical properties appear to have the greatest impact on the detected Cherenkov signal, while changes in average beam energy and angular distributions have smaller effects. Although field size–dependent changes in linac dose delivery influence the underlying production mechanisms, the relatively small changes in Cherenkov production per dose suggest that observed field-size dependence is primarily related to optical transport under the conditions investigated.

## CRediT authorship contribution statement

**Aubrey E. Parks:** Writing – review & editing, Writing – original draft, Methodology, Investigation, Formal analysis, Data curation, Conceptualization. **Wesley S. Culberson:** Writing – review & editing, Resources, Methodology. **Brian W. Pogue:** Writing – review & editing, Supervision, Resources, Methodology, Funding acquisition, Conceptualization.

## Declaration of competing interest

The authors declare the following financial interests/personal relationships which may be considered as potential competing interests: Brian W Pogue discloses financial involvement with DoseOptics LLC. Authors Aubrey E Parks and Wesley S Culberson declare that they have no known competing financial interests or personal relationships that could have appeared to influence the work reported in this paper.

## Appendix A Supplementary data

Supplementary material: Supplementary data provides information on Cherenkov photons generated from contaminant positrons and positrons, the interaction types for Cherenkov generation, and Cherenkov producing particle origins.
