# Supplementary material for: Assessment of Linac photon dose delivery on Cherenkov production
Source: Phys Imaging Radiat Oncol. 2026 Sep 15;42:101083. doi: 10.1016/j.phro.2026.101083 (PMC13599652; doi:10.1016/j.phro.2026.101083)
Supplement: Supplementary file 1 [file mmc1.docx]

**Supplementary Data**

Using Monte Carlo simulations, the photon and electron energy spectra are displayed in Figure S1. With a decrease in field size, there is a decrease in the fluence of low-energy incident particles due to preferential absorption in the linac head from increased collimation. This results in beam hardening from an increase in the average energy of the incident particles.

**Figure S1** (a) Linac photon spectra for a subset of field sizes investigated. (b) Corresponding electron fluence spectra for the same field sizes used. (c) Normalized photon spectra. Smaller fields exhibit decreased fluence with low energy photons. (d) Normalized electron spectra. Smaller fields exhibit decreased fluence with low energy electrons.

The total number of Cherenkov photons produced is shown in Figure S2a. The number of photons increases with both field size and depth, corresponding to the dose rise in the build-up region. Most Cherenkov photons originate from secondary electrons, as illustrated in Figure S2b, where their contribution closely matches the total photon production. Figure S2c further shows that Cherenkov photons from secondary electrons follow a similar trend, with larger fields generating more photons due to increased electron contamination. Although contaminant electrons still make a notable contribution, their photon yield is roughly an order of magnitude lower than that from secondary electrons.

**Figure S2** (a) Total number of Cherenkov photons produced. (b) Cherenkov photons produced due to secondary electrons. (c) Cherenkov photons produced due to electron contamination. (d) Cherenkov photons produced due to photons from pair production. (e) Cherenkov photons produced due to positron contamination.

In addition to secondary and contaminant electrons, positrons generated through pair production and contaminant positrons are also charged particles capable of producing Cherenkov photons in biological tissue. As shown in Figures S3a and S3b, the Cherenkov photons from these positron sources follow a trend similar to that observed for secondary and contaminant electrons, with photon yield increasing as field size increases. However, Figures S3c and S3d demonstrate that their relative contribution to the total Cherenkov production remains small, below ~2% for pair-production positrons and below ~0.25% for contaminant positrons, rendering their impact on field-size dependence negligible. Finally, when normalized to dose (Figures S3e and S3f), no additional field-size–dependent trends are observed that would significantly influence the overall Cherenkov signal.

**Figure S3** (a) Cherenkov photons produced from positrons from pair production. (b) Cherenkov photons produced from positron contamination particles. (c) Percent of Cherenkov created due to positrons from pair production. (d) Percent of total Cherenkov produced from positron contamination. (e) Cherenkov created from positrons per unit total dose. (f) Cherenkov created from positron contamination per unit total dose.

The number of particles responsible for generating Cherenkov photons follows a trend similar to that of the total Cherenkov photon yield (Figure S4a). As shown in Figure S4b, most Cherenkov-producing particles are secondary electrons, with additional contributions from contaminant electrons (Figure S4c). Positrons from pair production and contaminant positrons also contribute minimally, as illustrated in Figures S4d and S4e, respectively. Across all particle types, the results consistently show an increase in Cherenkov producing particles with larger field sizes at all depths, as well as with increasing depth.

**Figure S4** (a) Total number of particles that create Cherenkov. (b) Total number of secondary electrons that create Cherenkov. (c) Total number of electron contamination particles that create Cherenkov. (d) Total number of positrons from pair production that create Cherenkov. (e) Total number of positron contamination particles that create Cherenkov.

When the number of Cherenkov-producing particles is normalized to dose (Figure S5), the resulting gradient is shallower than for the total Cherenkov photons or unnormalized particle counts. This arises because dose deposition increases with depth, while the number of Cherenkov-producing particles grows. The total Cherenkov producing particles per dose (Figure S5a) and Cherenkov producing secondary electrons per dose (Figure S5b) exhibit similar trends. In contrast, the contaminant electron contribution per dose (Figure S5c) remains more stable with depth, reflecting the decrease in contaminant electrons with an increase in depth. Finally, the positron (Figure S5d) and contaminant positron (Figure S5e) contributions remain relatively constant with depth and are orders of magnitude lower than the secondary electron contribution.

**Figure S5** (a) Total number of particles that create Cherenkov per unit dose. (b) Number of secondary electrons creating Cherenkov per unit dose. (c) Number of electron contamination particles that create Cherenkov per unit dose. (d) Number of positrons from pair production that create Cherenkov per unit dose. (e) Number of positron contamination particles that create Cherenkov per unit dose.

When looking at the entire beam profile along the center and not only the most superficial centimeter, Table S1 displays the percentage of interactions that are responsible for creating the Cherenkov photons. Overall, ~96% of Cherenkov photons are created through Compton scattering, with additional contributions from primary particles, pair production, the photoelectric effect, and electron ionization. However, there are no main trends with changes in field size.

| **Field Size** | **Primary** | **Compton** | **Pair Production** | **Electron Ionization** | **Photoelectric Effect** |
| --- | --- | --- | --- | --- | --- |
| **0.5** | 0.127% | 95.875% | 3.194% | 0.793% | 0.011% |
| **1.5** | 0.080% | 95.778% | 3.327% | 0.805% | 0.010% |
| **5** | 0.089% | 95.773% | 3.313% | 0.816% | 0.009% |
| **10** | 0.134% | 95.807% | 3.246% | 0.802% | 0.011% |
| **15** | 0.167% | 95.816% | 3.211% | 0.795% | 0.011% |

**Table S1** The percent each interaction type is responsible for creating a parent particle

Additionally, Table S2 displays the percentage of the total number of Cherenkov creating particles that were created in each location. The majority (99%) of the Cherenkov creating particles are created within the water phantom itself, which is intuitive because most Cherenkov photons are created from secondary electron interactions. However, there are additional contributions to the Cherenkov created from contaminant particles, primarily from the World geometry, which includes both primary histories and charged particles created in the air column between the linac head and the phantom, which is consistent with literature.

| **Field Size** | **Base Plate** | **X Jaws** | **Y Jaws** | **MLCs** | **Exit Window** | **World** | **Phantom** |
| --- | --- | --- | --- | --- | --- | --- | --- |
| **0.5** | 0.31% | 0.01% | 0.02% | 0.02% | 0.03% | 0.31% | 99.29% |
| **1.5** | 0.01% | 0.00% | 0.01% | 0.05% | 0.03% | 0.26% | 99.64% |
| **5** | 0.00% | 0.01% | 0.02% | 0.03% | 0.03% | 0.28% | 99.64% |
| **10** | 0.00% | 0.02% | 0.02% | 0.02% | 0.03% | 0.32% | 99.58% |
| **15** | 0.00% | 0.02% | 0.03% | 0.02% | 0.03% | 0.35% | 99.55% |

**Table S2**: The percentage of the total Cherenkov-producing particles that are created in each geometry component of the linac head.
